# Nutritional considerations for alpha-gal syndrome: a mini-review

**DOI:** 10.3389/falgy.2026.1904888

**Published:** 2026-08-17

**Authors:** Abbigail Pace, Heather Norman-Burgdolf, Anna Cason

**Affiliations:** 1 Department of Dietetics and Human Nutrition, Martin-Gatton College of Agriculture, Food and Environment, University of Kentucky, Lexington, KY, United States; 2 Family and Consumer Sciences Extension, Martin-Gatton College of Agriculture, Food and Environment, University of Kentucky, Lexington, KY, United States

**Keywords:** alpha-gal syndrome, food allergies, medical nutrition therapy, nutrition assessment, registered dietitian nutritionist

## Abstract

Alpha-gal syndrome (AGS) is an immunoglobulin E-mediated sensitivity to galactose-alpha-1,3-galactose (“alpha-gal”), an oligosaccharide found in non-primate, mammalian-derived foods and products as well as some plant-based ingredients. Sensitization to alpha-gal is most commonly caused by a tick bite. Once sensitized, individuals experience delayed allergic reactions, typically 2–8 h after exposure to mammalian foods and other alpha-gal containing products. Symptoms range from urticaria to anaphylaxis and differ from exposure to exposure in the same individual, distinguishing AGS from other IgE-mediated food allergies. Recognition of AGS has grown since 2008, when patients receiving the anticancer drug, cetuximab, experienced severe reactions later linked to alpha-gal. In 2009, 24 cases were documented when patients endorsed anaphylaxis and urticaria several hours after eating meat. Today, an estimated 15,000 new cases occur annually in the United States, with many more suspected due to testing limitations and limited awareness among affected individuals and healthcare professionals. AGS has been diagnosed on every continent except Antarctica though there is not a global estimate of AGS prevalence at this time. Given the varying types and severities of sensitivity of this IgE-mediated food allergy, registered dietitian nutritionists (RDNs) are vital members of the care team. This mini review provides an overview of AGS for nutrition professionals that are responsible for delivering medical nutrition therapy, nutrition education, and nutrition counseling across clinical, community, and food service settings.

## 1 Introduction

Galactose-alpha-1,3-galactose (also known as “alpha-gal”) is an oligosaccharide commonly found in glycoproteins and glycolipids in mammalian cells, tissues, and fluids, but not in humans or other old-world primates (1). For most individuals, ingestion of alpha-gal is unproblematic; however, an increasing number of individuals develop sensitivity following bites from 9 different tick species depending on geographical area (2, 3). In sensitized individuals, IgE antibodies form in response to tick saliva containing alpha-gal, prompting the immune system to perceive it as a foreign antigen which initiates innate and adaptive responses (4). Emerging evidence indicates ticks may synthesize alpha-gal endogenously while experts previously believed ticks acquired alpha-gal from feeding on mammalian hosts (3, 5, 6). Ongoing research aims to clarify how these pathways contribute to sensitization and the development of AGS.

With ticks as the primary mechanism for acquiring the syndrome, individuals who spend considerable amounts of time outside in tick-prone habitats for their occupation or recreation are at greatest risk of developing AGS. For example, it was reported that forestry workers are two times as likely to develop AGS compared to the general population (7). Other groups with increased risk include farmers, ranchers, hunters, landscapers, individuals with hobbies that include substantial time outdoors, a previous history of food allergies, previous Lyme disease infection, and individuals with A or O blood types (8, 9).

It is possible for an individual to experience different levels of reaction depending on the type and amount of mammalian product consumed with products high in fat as the most likely to cause a severe reaction (10). Abdominal cramping, urticaria, and anaphylaxis are common (11). There is a group of individuals that present with gastrointestinal distress phenotype meaning presentation can resemble irritable bowel syndrome which may lead to delayed diagnosis of AGS (12).

The delay in the presentation of symptoms is a hallmark of AGS. Symptoms can take up to 2–8 h following ingestion of an alpha-gal containing food or product to appear, frequently after 10pm at night (10, 13, 14). Recent studies have proposed that this delay is related to lipid metabolism as alpha-gal is incorporated within a glycolipid (15). The ingested food particle undergoes intestinal emulsification, absorption, and is re-packaged into a chylomicron (16). The chylomicron is transmitted into the circulatory system via the thoracic duct. At this point, histamine and other immune system mediators produce the sensitivity reaction (10). These challenges are compounded by the limited awareness among healthcare providers concerning AGS and its symptoms which can delay diagnosis (17).

To obtain an AGS diagnosis, a knowledgeable physician or allergist collects details concerning medical history, onset of sensitivity reaction, dietary patterns, signs and symptoms of a sensitivity reaction, timing of symptom onset, and changes to symptoms after initiation AGS elimination diet. A positive blood test of serum alpha-gal IgE (>0.1 IU/mL alpha-gal sIgE) confirms the diagnosis within the context of the information above (10, 14, 18). It should be noted that levels of IgE are not correlated with severity of reaction, however IgE levels are needed for diagnosis and can be informative in a clinical context, including recency of AGS development. Rather, the quantity of tick bites and the timing of the most recent bite are better predictors of reaction severity (10). While the oral food challenge is the gold standard for diagnosis of most IgE-mediated food allergies, there are limitations to this diagnostic technique due to delayed reactions after ingestion (10). Skin Prick Tests are unreliable in the diagnosis of AGS due to the variations in levels of sensitivity (10). If required, these diagnostic tests should be completed in a specialized facility under medical supervision due to the risk of anaphylaxis.

## 2 Methods

The purpose of this mini-review is to provide information within an underexplored context, specifically nutrition considerations of AGS. To the authors' knowledge, there are limited published articles concerning this topic beyond recommendations of elimination or restriction of problematic foods and products. Therefore, it is pertinent to offer an overview for nutrition professionals that practice medical nutrition therapy, nutrition education, and nutrition counseling across clinical, community, and food service settings. This review was modeled after published articles that presented clinical information applicable to other professions (15, 19–21).

PubMed and Google Scholar were searched for relevant published articles utilizing terms, including “nutrition”, “nutrition considerations”, “nutrition implications”, and “diet” for “alpha-gal syndrome”. As information was scarce, authors expanded to include key articles for the RDN's role in management of food allergies. This information was applied to the AGS context by the authors that have expertise as Registered Dietitian Nutritionists with specializations in clinical nutrition, nutrition counseling and education, and Cooperative Extension.

## 3 Nutrition care process

### 3.1 Nutrition assessment

There is little guidance in the literature concerning the nutrition assessment of an individual with AGS. Procedure for nutrition assessment is similar to other IgE-mediated allergies (22, 23). A comprehensive nutrition assessment is required to assess client history; food/nutrition related history; anthropometric measurements; biochemical data, medical tests, and procedures; and findings from nutrition-focused physical exam. Venter et al. (23) outlines how RDNs should inquire about the history of allergic reactions, time of onset, symptoms, and duration of symptoms. It is possible for reactions to change over time (10). A food diary should be used to help identify specific trigger foods/products, symptoms, and timing of symptoms onset. Patients may react to certain foods at one time and not react to them later. RDNs should ask patients to monitor symptoms and reactions, then report pertinent information to the care team. In addition to symptoms, anthropometric data and results of nutrition-focused physical exams should be assessed for abnormalities in nutrition status and growth trends (22, 24, 25).

It is important to ask about medications, supplements, and other products that are consumed by the patient. Infant, enteral, parenteral nutrition formulas, nutrition supplement ingredients, and medical equipment should be reviewed and assessed for inclusion of ingredients or products containing alpha-gal (26). It should be noted that intravenous administration of alpha-gal containing medicines causes an immediate reaction. Therefore, RDNs should work closely with pharmacists to monitor ingredients of parenteral nutrition components (4).

When intake is impacted, RDNs should assess the patient for malnutrition and nutrient deficiencies, particularly with the GI distress phenotype. Symptoms such as abdominal pain, nausea, vomiting, and diarrhea are common and can significantly impact nutrition intake and absorption of nutrients (12). Dietary intake and biochemical data should be evaluated regularly to monitor for possible nutrient deficiencies, such as iron, B12, thiamin, riboflavin, calcium, and vitamin D (24, 25). Bone density assessment may be required in severe restrictions that exclude dairy and other foods containing calcium and vitamin D (27).

RDN assessment should include questions about the frequency and duration of exercise, use of non-steroidal anti-inflammatory drugs (NSAIDs), and level of alcohol intake as reactions to alpha-gal can be intensified with these cofactors (28). In general, the action of cofactors on severity of allergic reactions is poorly understood (29). However, it is suggested that these cofactors may increase GI absorption of the allergen as a result of increased intestinal permeability from exercise and NSAID use or relaxation of gut epithelium following intake of alcohol (29). There are other mechanisms, such as an increase in blood circulation or increased immune system reactivity, that can be direct effects of cofactors.

### 3.2 Nutrition diagnosis

Nutrition diagnoses should be developed appropriately for the individual patient. Possible nutrition diagnoses may include but are not limited to inadequate energy intake; inadequate protein intake; inadequate vitamin intake; inadequate mineral intake; altered GI function; unintended weight loss; underweight; malnutrition; food and nutrition related knowledge deficit; disordered eating pattern; impaired ability to prepare foods/meals; poor nutrition quality of life; and self-feeding difficulty (30).

### 3.3 Nutrition intervention

There is no cure for AGS, and guidance on food interventions is limited beyond elimination or restriction of foods and products that cause a reaction. The literature does offer advice on the RDN’s role for management of food allergies which can be applied to this context (31). Leone et al. (32) noted the role of the RDN includes offering guidance regarding the elimination of problematic foods; providing education on reading and interpreting food labels; providing a detailed list of substitutions that can support nutrition adequacy; identifying, preventing, and treating any nutritional deficiencies; and providing recommendations on nutrition supplements, if warranted, as supplements may contain sources with alpha-gal.

#### 3.3.1 Dietary modifications

**Figure 1:**
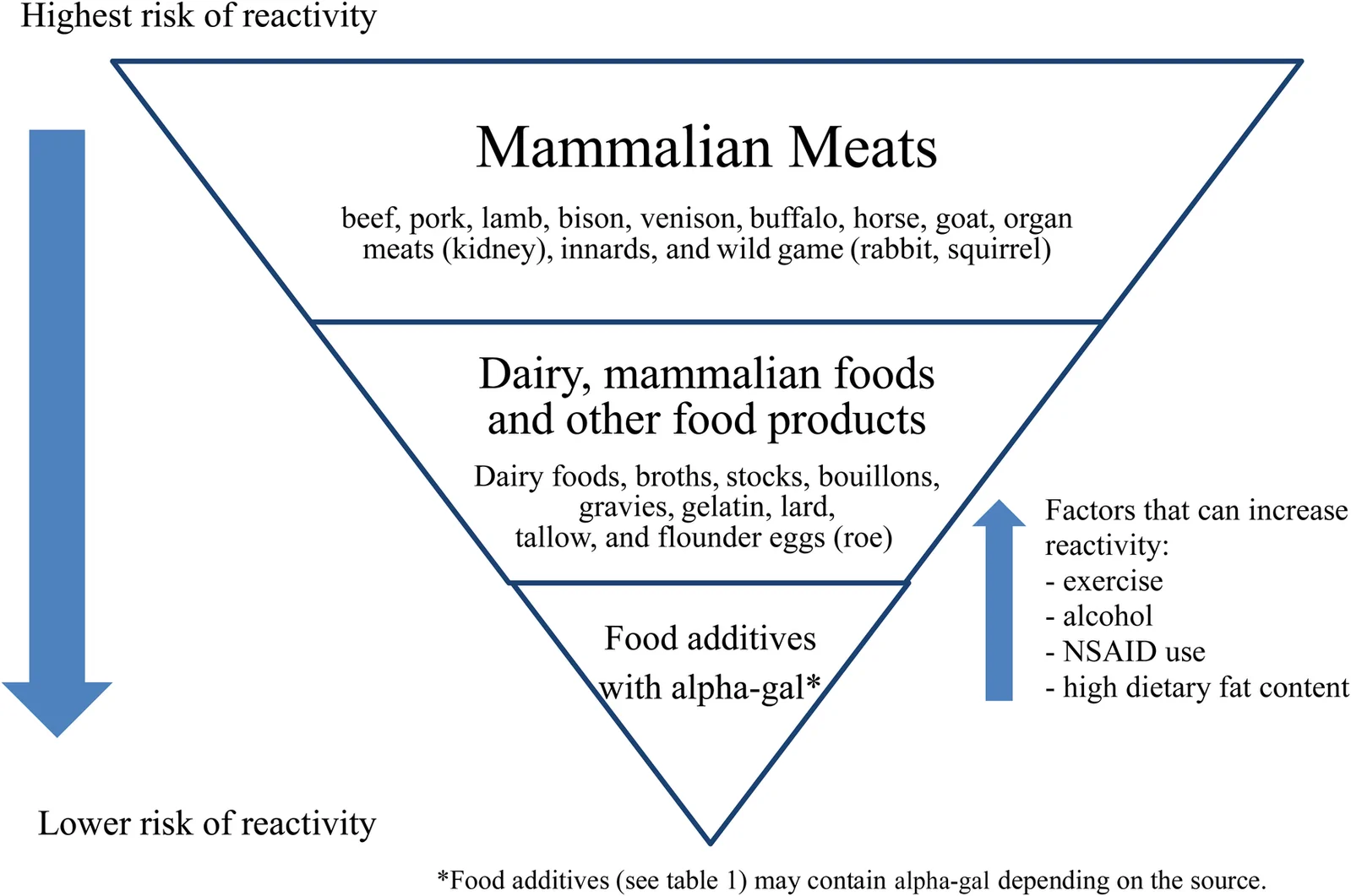
Tiered avoidance or elimination strategy based on highest and lowest reactivity (10, 28).

Most importantly, management of AGS should be individualized. Individuals sensitized to alpha-gal can take a tiered approach to elimination of foods and food products that contain alpha-gal. At new diagnosis of AGS, removal of mammalian meats is recommended, particularly with cuts of meat with high fat content or cooking with high fat methods, as it is most likely to lead to a reaction (see Figure 1) (10, 28, 33). This most commonly includes beef, pork, lamb, bison, venison, buffalo, horse, goat, organ meats (kidney), innards, and wild game (rabbit, squirrel), particularly foods or cuts of meat with a higher fat content. Dairy foods, broths, stocks, bouillons, gravies, gelatin, lard, tallow, and flounder eggs (roe) (34) contain alpha-gal and should be removed if symptoms continue to occur after the removal of mammalian meats (10). Other food additives and ingredients that contain alpha-gal, such as carrageenan, glycerin, and gelatin (see Table 1), should be removed if reactions continue after the removal of foods and products in tiers 1 and 2 (10). Highly sensitized individuals may need to remove all tiers if reactions are present. Timing between progression to the next tier should be the subject of future clinical studies.

**Table 1:** Common food additives that contain alpha-gal (10).

Common food additives that contain alpha-gal			
Arachidonic acid	Caseinate	Lanolin	Stearic acid
Arachidyl propionate	Castoreum	Lard	Sodium caseinate
Bovine extract	Collagen	Magnesium Stearate	Tallow
Carrageenan	Gelatin	Myristic acid^a^	Whey
Casein	Glycerin^a^	Oleic acid^a^	

a Products may be mammalian-derived or plant-sourced.

Nutrition education should support identifying foods and ingredients that contain alpha-gal on food labels and ingredients lists. Food manufacturers may change ingredients in a packaged product without notice. RDNs should emphasize simple, sustainable label review practices and be mindful to avoid inducing anxiety or obsessive habits. Additional tactics for managing severe AGS include evaluation of food claims and labeling. Some products labeled “plant-based”, “vegetarian”, or “vegan” may be safe for an individual with AGS, though these terms are not a guaranteed safety net. Third-party vegan certification labels may be a more reliable indicator that a product is free of animal-based products but should not be the only form of label reading used. It is important for patients to be able to identify any “safe” foods that may use mammalian casings or packaging. For example, chicken sausage that utilizes pork casing could cause a reaction in an individual with AGS (10). RDNs should be well-versed in food label regulations in the area in which they practice to best educate patients on appropriate reading of food labels and claims.

Cooking does not destroy alpha-gal in foods and can increase antibody binding and immune recognition (35). An allergic reaction can occur through inhalation or exposure to fumes, such as smoke or steam, from cooking mammalian meat. However, no studies have been published to identify the route of exposure (10). As with any food allergy, food safety practices are important to prevent a reaction (22). Appropriate measures to prevent cross-contamination during the preparation, storage, or serving of foods should be practiced by keeping safe and unsafe foods separate, cooking in separate dishes with utensils designated for safe foods, and plating foods separately.

RDNs should encourage a balanced diet with modification to the foods and products to avoid reactions. Proteins that do not contain alpha-gal, such as poultry (chicken, turkey), eggs, seafood, beans and lentils, nuts, seeds, and whole grains are safe to consume. Emu, ostrich, and duck are also safe to consume though less common and difficult to access. RDNs can educate patients on consuming complete sources of protein to meet requirements for essential amino acids, B12, vitamin D, calcium, and iron to reduce risk of nutrient deficiencies.

Patients should also be cautioned against following a restrictive diet, like a vegetarian or vegan diet, without proper guidance. If excluding dairy or animal-based foods and products, micronutrient levels should be monitored with review of dietary intake, physical symptoms of nutrient deficiency, and biochemical indicators prior to the nutrient supplementation. These micronutrients include vitamin B12, iodine, iron, choline, vitamin D, and calcium, depending on what foods are restricted (36). Individuals that utilize plant-based alternatives, like dairy alternatives from nuts and seeds, should be cautious of carrageenan as it is commonly used as a thickener and stabilizer in these products and may cause a reaction in alpha-gal sensitive individuals (10).

Management of AGS in healthcare and food service settings is comparable to other food allergens (37). Healthcare, food service, and school staff should be trained in management of food allergens to avoid cross contamination, accidental exposure, hidden ingredients, and emergency protocol for exposure. Alpha-gal should be listed as a drug allergy and a food allergy in health records. Individuals with severe allergies may experience symptoms from exposure to alpha-gal in a variety of clinical treatments including gelatin-containing vaccines, heparin, porcine-derived thyroid hormones, pancreatic enzymes, bioprosthetic heart valves, and antivenom (10, 26). Individuals with sensitivity to AGS should inform staff, provide emergency protocol plans, and carry epinephrine as recommended by their medical team.

It is possible for immune response to alpha-gal to diminish or change overtime without subsequent tick bites (3). To date, there is one published study that implemented a successful protocol for reintroduction of alpha-gal with gradual exposure to fat content and increased quantity of beef over 9 days (38). Inclusion criteria for this study were very strict; therefore, the sample was limited to 19 patients with declining alpha-gal sIgE values. All participants had reintroduced and tolerated dairy before the start of the exposure. Participants began with consuming lean mammalian meat and progressed to fatty mammalian meat over 9 days. All 19 participants successfully completed the inclusion and were able to move to a fully liberalized diet. This data is preliminary, and more research is needed before application to clinical practice with the guidance of appropriately trained medical professionals. Reintroduction of problematic foods should not be attempted without the guidance or supervision of medical professionals, particularly if an individual has a history of systematic reactions or anaphylaxis.

### 3.4 Monitoring and evaluation

Patients with a new diagnosis of AGS may require more frequent follow-up upon initiation of an AGS-safe diet. RDNs should regularly evaluate changes or patterns of nutrient intake, food and product restrictions, symptoms, symptom severity and frequency, and presence of cofactors. Height, weight, and lean body mass in adults along with growth trajectory in children should be monitored for signs of malnutrition. Depending on the level of restriction, biochemical labs, particularly for micronutrients, should be followed for deficiencies. RDNs should use appropriate clinical judgement for monitoring nutrition diagnoses, interventions, and outcomes. RDNs should work within a multi-disciplinary team of medical and mental health professionals and request referrals when necessary.

#### 3.4.1 Referrals

Dietary management is one pillar of AGS treatment. Allergists, immunologists, or primary care providers lead the team in AGS treatment. Gastroenterologists, pharmacists, dermatologists, and other specializations may be appropriate referrals depending on symptoms. AGS management often involves changes to lifestyle factors that affect the individual and their families, requiring additional referrals. It is important for RDNs to work closely with these providers to support patient care as outlined above.

Food allergy management can significantly impact social functioning and quality of life of patients and their caregivers due to the rapid onset and often delayed diagnosis of AGS (39, 40). A referral to a mental health specialist can help mediate the anxiety of food allergy management. Additional management strategies to alleviate the mental health burden of food allergy management, as noted by Casale et al. (39) include proper education and training on using epinephrine autoinjectors, availability of emergency medical services, connections to support groups, and discussions between parents/caregivers of children with AGS and school officials.

Additionally, the cornerstone of AGS treatment is avoidance of subsequent tick bites (5, 41, 42). RDNs can discuss the importance of proper protection from tick bites to avoid subsequent bites. RDNs may refer patients to local health departments or experts for education and resources on tick education. Additionally, public health organizations and specialists in that field, like entomologists, can be helpful in education and obtaining appropriate resources.

## 4 Conclusion

AGS is an emerging and complex condition with serious nutrition implications, and RDNs play an essential role helping individuals manage it with clarity and confidence. Care should be individualized, with a tiered approach to avoidance that helps patients reduce reactions without diving into unnecessary restriction. RDNs can support balanced intake by encouraging the continued use of safe foods, monitoring for nutritional adequacy when dairy or multiple animal-based foods are removed, and helping patients understand how cofactors like exercise, NSAIDs, alcohol, and subsequent tick bites may influence reactions. Collaboration with allergists, attention to emergency preparedness for those with severe reactions, and guidance on tick-bite prevention are also important components of care. At the same time, many gaps remain in the evidence base, including the prevalence of micronutrient deficiencies in AGS, the psychosocial burden of navigating hidden ingredients and labeling, and the best protocols for reintroducing mammalian foods and ingredients when IgE levels decline. As research continues to evolve, RDNs will remain central in helping individuals with AGS maintain nutritional adequacy, reduce reactions, and feel supported in both the practical and emotional aspects of living with a complex food allergy.
